# Therapeutics of the Throat: Rhus Toxicodendron

**Published:** 1888-04

**Authors:** C. E. Chase

**Affiliations:** Utica, N. Y.


### THERAPEUTICS OF THE THROAT.

### RHUS TOXICODENDRON.

### OBJECTIVE.

Cellulitis of neck.

THROAT MUCH SWOLLEN EXTERNALLY, AS IF THE PAROTID AND SUBMAXILLARY GLANDS WERE GREATLY ENLARGED.

PAROTID AND SUBMAXILLARY GLANDS HARD AND SWOLLEN.

Tonsil (right) covered with yellow membrane. Erysipelatous inflammation of throat.

Saliva bloody; runs out of the mouth during sleep.

*Tonsils, especially the right one, swollen, red, and partly covered with slough-like membrane.*

Tongue, *dry, red, cracked; has a triangular red tip.*

Tongue white, often on one side; *yellowish;* covered *with brown mucus;* takes imprint of teeth.

*Redness of the fauces.*

Saliva runs out of mouth during sleep.

Much tough mucus in mouth and throat.

### SUBJECTIVE.

Mouth dry with much thirst.

*Sensation of dryness in throat.*

Roughness in throat and trachea, as if chest were raw and sore; provokes a hacking cough.

Soreness of throat with intense burning extending to stomach —as from an internal swelling, with bruised pain; also when talking, with pressure and stinging when swallowing; *deglutition difficult with stitching pain,* and rawness in left tonsil, when swallowing, at the root of the tongue—of the larynx; feels stiff after straining the throat.

Whole pharynx feels stiff and bruised and is involved in erysipelatous inflammation; hoarseness after straining the throat.

Burning in the throat; in the œsophagus, in the forenoon.

Sticking or stinging pain in the tonsils, worse when beginning to swallow; whenever the throat is dry there is sticking on swallowing. Pricking in throat like needles, low down on sternum.

Stitch in throat on swallowing or yawning, violent, as though she had swallowed a needle; violent stitches that commence dull and end sharp and pointed, in region of epiglottis, when not swallowing, always relieved by swallowing.

Feeling of swelling, with bruised pain; erysipelatous inflammation; cellulitis; drowsiness.

Swelling of submaxillary glands, with sticking pain on swallowing.

Sensation in pit of throat as if stopped and constricted in trachea, better for a short time by eating and drinking.

Difficulty of swallowing from soreness and apparent smallness of the throat. Difficult swallowing of solids, as from contraction.

Œsophagitis after corrosive substances.

She is unable to drink, as every swallow chokes her as if the pharynx were inactive or paralyzed, associated with a sensation of dryness in throat posteriorly.

Tenacious mucus in throat, disappears after hawking a little, but leaves a roughness.

Profuse hawking of mucus in the morning; much mucus in throat and posterior nares.

Pressure in throat when swallowing, less on swallowing food than on empty swallowing.

Sensation of coldness in the throat on expiration as if the breath were cold.

Throbbing pain in the throat posteriorly.

Putrid breath.

### CONCOMITANTS.

*Anxiety and apprehension, with despondency, and great weakness; restless mood; irritability.*

*Low spirited, inclined to weep.*

*Heaviness and dullness of head, with great dizziness on rising as if intoxicated.*

Head very heavy, as of a weight in forehead; brain feels loose as if it hit against the skull when shaking it.

Pain and stiffness in nape of neck.

Sickly expression, face sunken, blue rings around eyes.

Tip of nose red and sore.

Hot burning beneath left nostril.

Frequent sneezing.

Nose bleed on stooping in the morning.

Profuse discharge of mucus from the nose in the morning.

Soreness in the nostrils.

Corners of mouth sore and ulcerated.

*Cramp-like pain in articulation of lower jaw close to the ear, during rest and motion of the part,* relieved by hard pressure and by warmth.

*Pressive and digging pain in the glands beneath the angle of the lower jaw,* even when at rest.

*Blisters on the tongue;* TONGUE SORE WITH REDNESS OF APEX.

Burning and smarting of tongue, with increased saliva.

GREAT THIRST, WITH DRYNESS OF MOUTH AND THROAT; unquenchable thirst, wants cold drinks, worse at night, from dryness of mouth.

DESIRE FOR COLD MILK.

*Sensation of dryness on the tip of the tongue.*

Tongue very dry, which provoked drinking.

Water accumulates in the mouth. Saliva runs from the mouth during the afternoon nap. Frequent spitting of very tenacious mucus.

Fetid breath.

Coppery taste in the mouth, and a scraping feeling low down in the throat.

Disgusting, bitter taste, with dryness of the mouth, frequently woke her at night.

Taste of blood in the mouth when coughing, though no blood is raised.

A tickling cough that causes dryness in the throat, especially in the evening; dry cough in the morning with soreness of the throat.

Inflammation of the larynx, extending from the fauces, with heat; soreness and sense of suffocation; hoarseness from straining the voice.

Very great weakness, especially on walking in the open air; weariness worse on sitting, relieved while walking.

Stiffness on rising from a seat, stiffness and soreness all over, passes off with exercise; aching all over the body as if in the bones; *stiffness and aching, bruised pain in small of back, when sitting or lying, better from motion or lying on something hard.*

*Great debility, paralytic weakness and soreness, especially when sitting and at rest.*

Great restlessness from internal uneasiness; *restlessness at night, could not stay in bed.*

*Sleeplessness, with restless tossing about.*

Constant chilliness.

Sensitive to cold air.

*Fever in evening, with shivering, headache, and pain in the limbs.*

Mild delirium, of business matters, and as if he were undergoing a severe exertion. Muttering.

### AGGRAVATIONS.

From getting wet; in cold wet weather; on beginning to move; when swallowing—stitching and stinging pains in throat, when beginning to swallow—sticking or stinging in tonsils—also whenever throat is dry; on drinking every swallow chokes her, as if throat were paralyzed; pressure—worse on empty swallowing. Aggravation evening and night—sleepless after midnight.

### AMELIORATION.

By continued motion—by warmth; violent stitches, relieved by swallowing; constriction of trachea, relieved by eating and drinking.

### REMARKS.

Rhus is pre-eminently a rheumatic remedy and many symptoms of the throat appear to be seated in the muscles of the part and are analogous to those of the larger muscular tissues. Its well-known typhoid proclivities, however, render it particularly applicable to cases of a catarrhal nature associated with a low febrile condition. Even in diphtheria it will occasionally be useful, as its swollen glands, putrid breath, bloody saliva, etc., indicate. In the sore throat of scarlatina it will find its greatest use, as in my experience Rhus has been more frequently indicated in that disease than any other remedy; with the throat symptoms given above, are associated a dark-red, rough, miliary, sometimes vesicular eruption, much more often than the smooth bright-red rash of Bell. The symptoms indicate its usefulness in stricture of the œsophagus, especially if caused by burns, and in paralysis of the throat muscles.

### DIFFERENTIATION.

Rhus touches many other drugs at various points; in throat diseases Baptisia resembles Rhus in its typhoid tendency, but the prostration of Bapt. is greater, with a drowsy, stupid condition and besotted look instead of the restless irritability of Rhus—the throat, too, is dark-red, with dark putrid ulcers and a marked painlessness, while all the discharges are very offensive. With Phyt. the pains in the head, neck, back, are more intense, and while the patient is restless, every motion aggravates and makes him moan with pain, he is so sore; the throat is dark-red or purplish, with ulcerated follicles and sensation of intense burning, as of a red-hot ball, and on swallowing intense pains shoot up into the ears.

Rhus has an œdema of the throat resembling Apis, but, as Farrington says, the resemblance is superficial and should be guarded against, as the two remedies do harm if given in immediate succession.

Compare Lach., Ailanth., Arum-tr., Bry., Caust., Sulph.

UTICA, N. Y. C. E. CHASE.

